# Percutaneous vertebroplasty for Langerhans cell histiocytosis of the lumbar spine in an adult: Case report and review of the literature

**DOI:** 10.3892/etm.2012.791

**Published:** 2012-11-01

**Authors:** FEI FENG, HAI TANG, HAO CHEN, PU JIA, LI BAO, JIN-JUN LI

**Affiliations:** Department of Orthopedics, Beijing Friendship Hospital, Capital Medical University, Beijing 100050, P.R. China

**Keywords:** percutaneous vertebroplasty, Langerhans cell histiocytosis, spine, adult

## Abstract

Langerhans cell histiocytosis (LCH) is extremely rare in the lumbar spine of adults. The radiological features typically manifest as vertebral tumors. The exact etiology of LCH remains unknown. Langerhans cells may cause local or systemic effects. The most frequent sites of these bony lesions are the skull, femur, mandible, pelvis and spine. To date, only 3 spinal LCH cases treated by percutaneous vertebroplasty (PVP) have been reported. The present study reports a case of LCH of the fourth lumbar vertebra (L4) in a 51-year-old male with a 10-day history of low back pain, limited waist motion and right lower limb numbness. The patient was treated using PVP. The use of PVP for treating LCH of the spine was successful. The present study provides an up-to-date literature overview of LCH.

## Introduction

Langerhans cell histiocytosis (LCH) in the lumbar spine of adults is uncommon (1,2). A variety of treatment modalities have been reported for the management of LCH of the spine, including conservative treatments, systemic chemotherapy, curettage (with or without bone grafting), internal fixation and fusion, percutaneous vertebroplasty (PVP), corticosteroid injection into the lesion and radiotherapy (3). Although the clinical results are largely satisfactory, there is not a defined therapeutic algorithm. In the present study, the case of a 51-year-old male with LCH of the fourth lumar vertebra (L4) is reported.

## Case report

**Figure 1:**
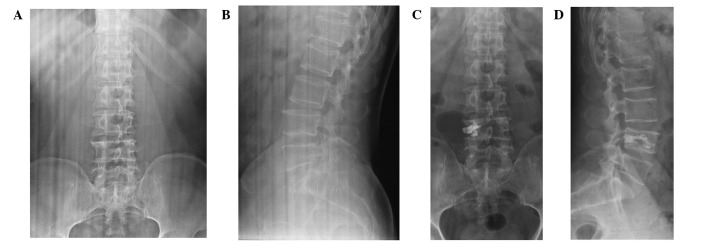
Plain radiographs of the lumbar spine, (A and B) preoperative and (C and D) 6 months postoperative.

**Figure 2:**
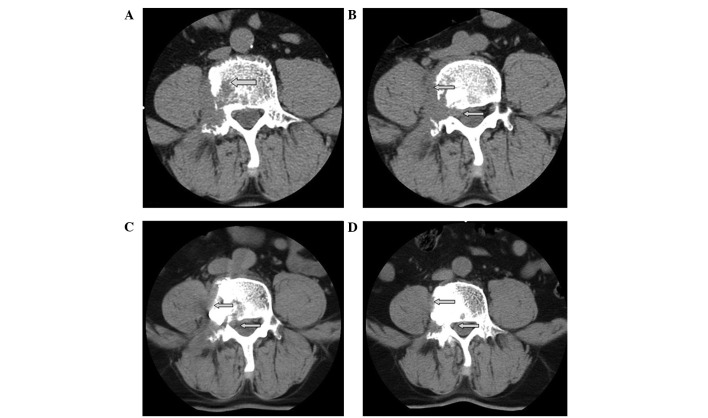
An osteolytic vertebral fracture in the right lateral mass of the L4 and accessories, accompanied by (A) a paravertebral and (B) intraspinal soft tissue mass. The mass (C) significantly decreased 5 days after PVP and (D, arrow) disappeared completely after 3 chemotherapy cycles. Computed tomography (CT) showed the distribution of bone cement in the osteolytic lesion of the L4 with minimal right lateral cement leakage. L4, fourth lower lumbar vertebra; PVP, percutaneous vertebroplasty.

**Figure 3:**
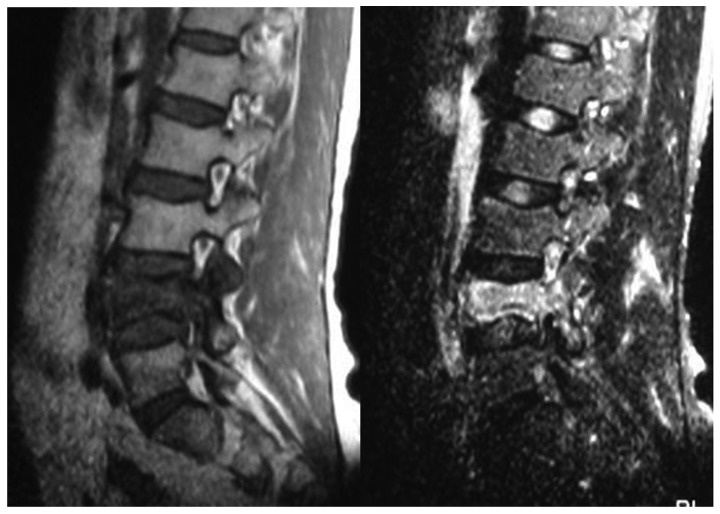
MRI, sagittal section. Hypointensity on T1-W images of L4 planar appearance with a mild compression fracture. T2-W images reveal more marked hypersignals of the body of L4 associated with the recent collapse. MRI, magnetic resonance imaging; L4, fourth lower lumbar vertebra, T1 W, T1 weighted; T2 W, T2-weighted.

**Figure 4:**
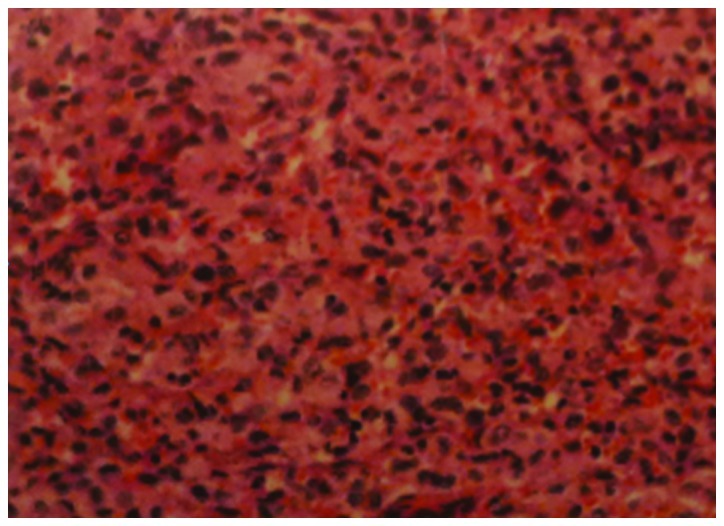
High-power photomicrograph shows Langerhans’ histiocytes and mixed numerous eosinophils. Neoplastic cells showing abundant eosinophilic cytoplasm, round to oval vesicular nuclei, and distinct nucleoli.

The 51-year-old male patient exhibited a 10-day history of low back pain, limited waist motion and right lower limb numbness. The patient reported no pain at other sites, exhibited no fever or night sweats and was unable to recall any recent injury. The patient’s past medical history was unremarkable for trauma or other bone diseases. A physical examination demonstrated localized tenderness and percussion pain over the L4 spinous process, restricted waist motion and numbness of the right leg. Laboratory tests, including full blood cell count, serum electrolytes, renal and liver function tests, erythrocyte sedimentation rate (ESR) and C-reactive protein (CRP), did not reveal any abnormalities. An X-ray revealed that the lesion was limited to the left lateral mass of the atlas, causing a potential instability (Fig. 1A and B). Computed tomography (CT) revealed an osteolytic lesion in the right lateral mass of the L4 and accessories, accompanied by a paravertebral and intraspinal soft tissue extension (Fig. 2A and B). Magnetic resonance imaging (MRI) revealed osteolytic destruction of the vertebral body associated with a mild compression fracture that exhibited hypointensity on T1-weighted (T1-W) images and hyperintensity on T2-weighted (T2-W) images (Fig. 3). On the basis of the radiological features of the lesion, there was a high possibility that the patient had a neoplastic lesion. However, the radiological features of the lesion were not sufficient to establish the diagnosis of LCH with certainty. A C-arm X-ray machine-guided needle biopsy of the vertebral body was performed and the histopathological diagnosis was LCH. Immunohistochemical staining was positive for CD1a and S-100 (Fig. 4). Further diagnostic evaluation included a bone scan, CT of the lungs, pituitary hormonal evaluation and brain CT and abdominal ultrasound evaluation. No other LCH infiltration was identified in the patient and the patient was treated as suffering from a single-system and single-site disease.

**Figure 5:**
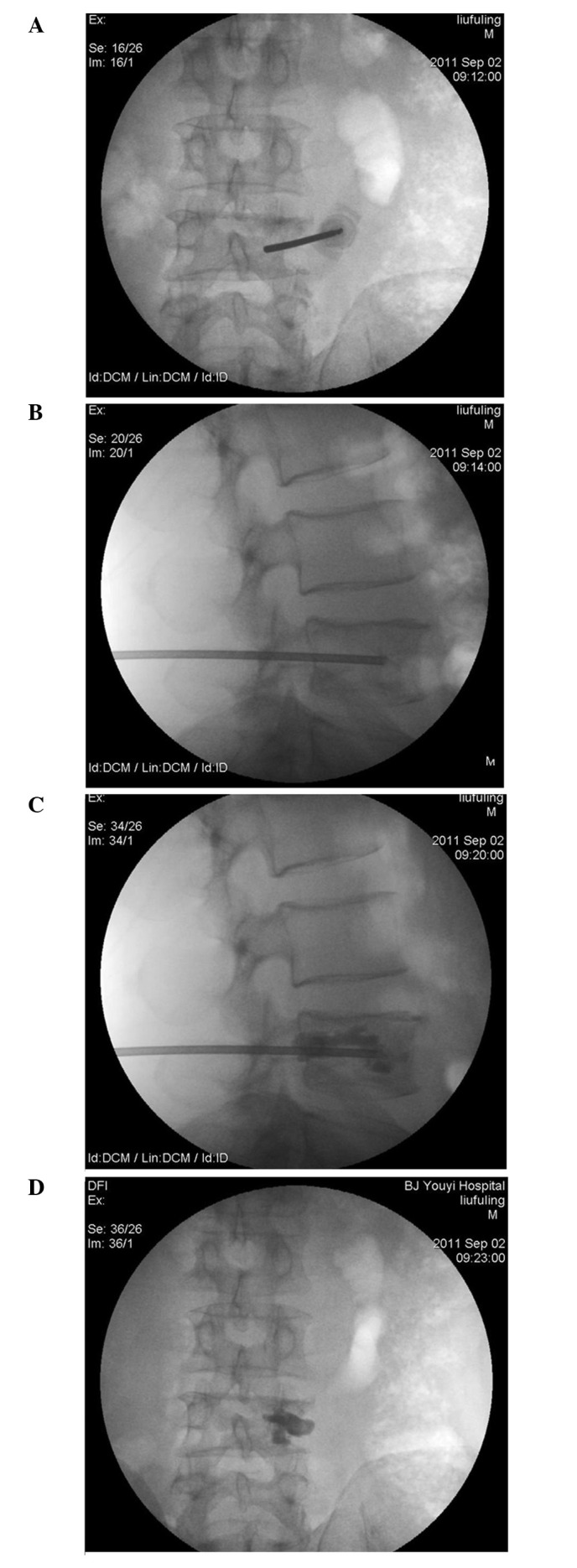
Radiography during PVP of the L4. Right posterolateral approach. Injection of polymethylacrylate and penetration of the L4. Images show cement filling at the right side of the vertebral body. PVP, percutaneous vertebroplasty; L4, fourth lower lumbar vertebra.

The patient underwent PVP (Stryker, Inc., Meyzieu, France) under local anesthesia in the prone position with the belly suspended in midair, under C-arm imaging guidance (Fig. 5). The amount of bone cement used to fill in the L4 was 3.6 ml. The blood loss during surgery was 5 ml. The spread of the cement was ideal with the exception of a small amount of paravertebral leakage of cement (Fig. 2C) which did not cause any symptoms. No complications were observed during the surgery or follow-up. After lying in bed for 6 h, the patient was able to sit freely and 24 h postoperatively, the patient was allowed to walk freely. Following the procedure, the low back pain was resolved completely and the patient’s neurological symptoms were rapidly alleviated and then gradually continued to be alleviated. The patient required the use of a weak opioid prior to the PVP but did not receive an analgesic afterwards. Notably, CT revealed a significant decrease in the paravertebral and intraspinal soft tissue extension 5 days after the PVP (Fig. 2C).

The patient received chemotherapy following PVP. The chemotherapy regime was 100 mg etoposide (days 1–3) and 60, 40 and 20 mg prednisone (days 1–7, 8–14 and 15–21, respectively) for 3 cycles. There were no serious side-effects of the chemotherapy. CT revealed that the paravertebral and intraspinal soft tissue extension disappeared after 3 cycles (Fig. 2D). The height of the vertebral body remained stable without further collapse and lumbar kyphosis did not occur. There was no recurrence and no other complaints over a 6-month follow-up period (Fig. 1C and D).

## Discussion

LCH is a rare disease associated with the proliferation of Langerhans cells (1,2). The incidence rate of LCH is approximately 1:1,500,000 (3). Although LCH mostly occurs during childhood, it may affect patients of any age from infants to elderly individuals. LCH is characterized by the clonal accumulation and/or proliferation of specific dendritic cells that resemble the normal epidermal Langerhans cell and are capable of infiltrating almost any organ (4). Although the cell of origin in this disease has now been defined, the exact etiology of LCH remains unknown. It is considered to be a neoplasm or infectious disease caused by a disorder during the immaturity of the immune system (5). LCH has 3 classic clinical syndromes that are considered to be variations of the same disease: i) eosinophilic granuloma; ii) Hand-Schüller-Christian disease; and iii) Letterer-Siwe disease (5).

The most frequent sites of the bony lesions of LCH are the skull, femur, mandible, pelvis and spine (3,6). LCH in the spine is reported to occur in between 6.5 and 25% of cases (7), with the most frequent site being the thoracic vertebrae (54%), followed by the lumbar (35%) and cervical (11%) vertebrae (5). Soft tissue extension has been reported in 50% of cases (6) and posterior arch extension in 65% (8).

The characteristic symptoms of LCH of the lumbar spine of adults are back pain, restricted range of motion and neurological symptoms, although neurological deficits are uncommon (9). Pain is explained by the onset of a collapse of the vertebral body with osteolysis. Neurological symptoms may be caused by the soft tissue extension. Spinal LCH is easy to misdiagnose as malignant tumors, lymphoma or tuberculosis. LCH should be included in the differential diagnosis of osteolytic and osteoblastic vertebral lesions. Although radiological studies and clinical characteristics may indicate the disease, these alone cannot result in a definitive diagnosis. Histopathological confirmation is essential. The histopathological diagnostic criteria require the expression of CD1a and S-100 antigen on the lesion cell surface for a definitive diagnosis (10).

There are various treatment modalities for LCH of the spine reported in the literature. Conservative measures are appropriate for mild isolated involvement of the spine without a risk of neurological damage or spinal instability, including simple observation, prolonged immobilization, nonsteroidal anti-inflammatory drugs or casting with or without initial bed rest (11–13). Open surgery should be reserved for patients with severe mechanical instability or deformity and/or neurological deficits caused by the compression (8,11). Due to the potential for secondary malignancy and vertebral growth-plate damage in the skeletally immature patients, radiotherapy appears to be overtreatment in isolated osseous cases (7,14,15). In cases where the patient is a child, radiotherapy may lead to the early closure of vertebral growth (16). Chemotherapy is suggested for treating disseminated LCH, such as multiple bone lesions or multi-system disease (3). It has been reported that chemotherapy is safe and effective for the management of LCH of the spine in patients with soft tissue extension (6) and may significantly reduce recurrence rates (17). Although these treatments were reported to produce satisfactory results with a recurrence rate of less than 20%, there has been no evidence suggesting that any one treatment is more advantageous than another (18–22).

PVP was developed by Galibert *et al*(23) and appears to offer an alternative to the preceeding treatments. The minimally invasive vertebroplasty apparatus consists of an introducing cannula, operative cannula, Kirschner guidewires, manual drill and reconstituted acrylic polymethylmethacrylate which is used to fill the vertebra via a transpedicular approach under C-arm imaging guidance. PVP is able to effectively relieve pain and strengthen the vertebra weakened by the disease, allowing spinal stabilization. PVP has been generally accepted as a safe and effective treatment option for patients with vertebral haemangioma (23), osteoporotic vertebral compression fractures (24) and spinal tumors (25). PVP is a new technique with a number of advantages; it is minimally invasive and does not require implants or open surgery and patients may recover rapidly. PVP is capable of relieving pain quickly and stabilizing the fracture by enhancing the rigidity and intensity of vertebra to allow early weight-bearing movements.

Only 3 cases concerning the treatment of LCH in the spine with PVP have been reported previously in the literature. Tan *et al*(26) performed PVP in a child with cervical LCH and the patient recovered well. Cardon *et al* used PVP in an adult with lumbar spine LCH and reported a good clinical result (27). Kevane *et al* performed PVP in an adult lumbar spine LCH case with marked symptomatic relief (28).

Although the mechanism of pain relief following PVP remains unclear, the majority of studies speculate that it may be due to: i) the heat generated during cement consolidation destroying the nerve endings in the surrounding tissues and killing tumor cells (29); ii) the injected bone cement improving the strength of the vertebral bodies and the stability of the spine, redistributing the mechanical forces, reducing the irritation to vertebral nerves (30,31); and iii) the cytotoxicity of the polymethylmethacrylate in the cement destroying nerve terminals and killing tumor cells (32–35).

In conclusion, when conservative treatments are not feasible and open surgical treatment is an overtreatment, PVP is a suitable alternative for treating patients with the progressive lesions of LCH in the spine and the potential risk of progressive vertebral compression fractures and neural compression, and may be new indicators of PVP. PVP relieves pain quickly and stabilizes the fracture of the vertebra with minimal invasion. Patients are able to recover rapidly and make early weight-bearing movements. Combination chemotherapy for treating the paravertebral and intraspinal soft tissue extension is safe and effective and may also reduce recurrence. Although the short-term results of PVP for LCH of the spine are promising, long-term follow-ups are essential for demonstrating the efficacy of PVP in cases of spinal LCH.
